# *Inter situ* collections as a strategy to conserve an exceptional plant species from the Amazon rainforest

**DOI:** 10.1371/journal.pone.0349107

**Published:** 2026-06-03

**Authors:** Diego Fernando Escobar Escobar, Markus Gastauer, Silvio Junio Ramos, Cecilio Frois Caldeira

**Affiliations:** Instituto Tecnológico Vale Desenvolvimento Sustentável, Rua Boaventura da Silva, 955 - Nazaré, Belém, Brazil; National Cheng Kung University, TAIWAN

## Abstract

*Ex situ* conservation is essential for achieving the goals of the United Nations Global Strategy for Plant Conservation and the Sustainable Development Goals. Although seed banking is the most common method of *ex situ* plant conservation, many ecologically and economically important species cannot be effectively conserved by this method (i.e., exceptional species). These species can be preserved through cryopreservation and *ex situ* living collections; however, cryopreservation is expensive and remains underdeveloped for many taxa, while *ex situ* living collections often have poor genetic representation and limited evolutionary potential. *Inter situ* collections—conservation plantings established outside the current species range under natural or semi-natural conditions—are well suited for conserving the genetic diversity of exceptional species and can complement *in situ* conservation. Here, we present a case study establishing and managing *inter situ* collections of an exceptional plant species from the Amazon rainforest, aiming to preserve its genetic diversity in the long term and establish new self-sustaining populations. In addition, the *inter situ* collection contributes to ecological restoration by enriching the understory layer in recovering forest areas where the collection was established. Our study provides a model for initiatives seeking to develop seed sources for further conservation actions, such as reintroduction or habitat restoration. The results highlight the importance of systematically monitoring propagule production and field establishment success at the individual level in order to continuously optimize seed harvesting and propagation strategies. Finally, we propose practical guidelines for conserving plant species through *inter situ* collections, intended as an entry point rather than a comprehensive framework. We hope these findings encourage stronger integration between *inter situ* and *in situ* conservation approaches for exceptional tropical plant species*.*

## Introduction

The rapid rate of land-use change significantly reduces biodiversity, simplifies species interaction networks, alters biogeochemical cycles, and diminishes the functionality and regenerative capacity of ecosystems [1–3]. These processes result in reduced climate regulation, soil degradation, rising global temperatures, and constrained water availability [4]. In this context of intense anthropogenic modification, *ex situ* and *in situ* conservation have emerged as key strategies to mitigate biodiversity loss and meet global targets, such as those established by the Global Strategy for Plant Conservation, which aims to conserve the genetic diversity of threatened plant species and crop relatives in *ex situ* collections [5].

The primary aim of conservation collections is to support species persistence and reduce extinction risk through the long-term preservation of genetic diversity and the provision of plant material for *in situ* conservation actions [6,7]. *Ex situ* conservation methods can be broadly classified according to the type of biological material conserved and its metabolic state. These approaches include the preservation of plant material in a low-metabolic state — such as seeds or plant tissues stored through seed banking or cryopreservation — and the maintenance of fully developed plants under active growth conditions in *ex situ* living collections. The most appropriate method depends on species life history and physiology, available propagation techniques, institutional capacity, and conservation objectives [6,7]. Seed banking involves the storage of desiccation-tolerant seeds at low temperatures, while *in vitro* culture and cryopreservation enable long-term storage of seeds, embryos, or other tissues in liquid nitrogen [8,9]. In contrast, *ex situ* living collections or field genebanks maintain whole plants under continuous cultivation and horticultural care in constructed landscapes outside their natural populations, typically in botanical gardens or research facilities [8,9].

Seed banking is the most widely used method of long-term *ex situ* conservation because it can preserve high levels of genetic diversity at relatively low cost and with minimal space requirements for long periods [10]. However, some species, known as exceptional species, cannot be conserved in this way due to the absence of viable seeds, sensitivity to desiccation, or short-lived orthodox seeds [11]. Conserving these species is essential for achieving the targets of the Convention on Biological Diversity (CBD), given that approximately 40% of threatened plant species fall into this category [12–14]. Furthermore, exceptional species are common in several plant families, including Poaceae, which contains approximately one-third of crop species, as well as highly diverse families such as Asteraceae and Orchidaceae [13,15,16].

*In vitro* methods and e*x situ* living collections provide alternatives to seed banking for conserving exceptional species [9,14,17]. However, *in vitro* conservation is expensive and remains underdeveloped for many species, while *ex situ* living collections often exhibit poor genetic representation and are highly vulnerable to genetic drift, artificial selection, and outbreeding depression [6,17,18]. As a result, the conservation value of many *ex situ* collections is limited by their inability to adequately represent genetic diversity or provide plant material capable of supporting effective *in situ* conservation actions [6,19–21].

*Inter situ* collections are considered an intermediate approach between *ex situ* and *in situ* conservation because they are established outside the original populations; however, unlike *ex situ* living collections, plants are cultivated under natural or semi-natural conditions to promote long-term persistence through the establishment of self-sustaining populations [22]. These collections may be established either outside the current species range but within its recent historical distribution [22], or beyond its historical range [19,23]. Conservation introductions are also considered a form of *inter situ* conservation, as new populations are established in the wild under environmental conditions similar to those of the original *in situ* populations [14,24].

Unlike *ex situ* living collections, *inter situ* collections are established in areas large enough to maintain the genetic diversity of a population or species [6,19]. The wild or semi-wild conditions in which these collections are maintained may enhance plant reproduction and recruitment, while also providing opportunities to improve our understanding of species’ reproductive biology and propagation requirements [6,19]. Moreover, *inter situ* collections allow threatened species to be used in the restoration of degraded habitats while simultaneously expanding their distribution beyond currently occupied sites [6]. Consequently, *inter situ* collections are increasingly used as a conservation strategy for threatened and exceptional species, as they support long-term species preservation, maintain evolutionary potential, and provide propagation material for *in situ* conservation efforts [25–27].

Here, we present a case study on the establishment of an *inter situ* collection across restoration areas using an exceptional plant species from Amazonian forests. The collection was established from seeds harvested in natural populations and involved three main steps: [1] preparation before collecting propagation material; [2] harvesting, post-harvest handling, and sapling production; and [3] establishment, maintenance, and long-term monitoring of the *inter situ* collection. The long-term goal of this initiative is to establish new self-sustaining populations outside the current species’ range that represent the genetic diversity of wild populations [23,26]. However, because the collection is still in its early stages, success was evaluated using short-term performance criteria — including the survival (number and percentage) of one-year-old juveniles [28], success at each propagation stage, the number of established juvenile plants (one- to three-year-old individuals), and the number of wild maternal lines represented in the collection. Additionally, based on this case study and a review of the literature, we propose introductory guidelines for establishing and managing *inter situ* collections aimed at preserving genetic diversity over the long term and providing seed sources for *in situ* conservation actions, such as reintroduction and habitat restoration (S1 File Supplementary guidelines).

## Materials and methods

### Preparations before collecting propagation materials

#### Why conserve *Pilocarpus microphyllus* genetic diversity at Carajás FLONA?.

*Pilocarpus microphyllus* Stapf ex Wardleworth, an endemic understory shrub of the eastern Brazilian Amazon known locally as jaborandi, is the only known natural source of pilocarpine, an alkaloid widely used in the treatment of diseases such as glaucoma, xerostomia, Sjögren’s syndrome, and presbyopia [29–31]. Furthermore, jaborandi has an important socio-economic role, as harvesting its leaves has provided a significant income for thousands of families [29]. However, *P. microphyllus* is currently listed in the Brazilian Red List as vulnerable due to significantly decline in population size and habitat quality, primarily driven by overharvesting and land-use changes such as agriculture, cattle ranching, and mining [30].

One of the largest remaining populations of *Pilocarpus microphyllus* occurs in the Carajás National Forest Natural Reserve (Carajás FLONA), which is a hotspot of genetic diversity for the species [29]. Although FLONA Carajás is a sustainable-use protected area, ongoing activities such as mining, leaf harvesting for pilocarpine production, and seed collection for restoration may pose cumulative threats to natural jaborandi populations. Therefore, *in situ* conservation of *P. microphyllus* genetic diversity within the Carajás FLONA is imperative. However, this effort must be complemented by *ex situ* conservation, given increasing land-use changes across the Amazon and the historical overharvesting of the species [3,30,31]. Integrating complementary conservation approaches can enhance long-term genetic preservation while ensuring a reliable supply of propagules to support species recovery.

### Determine the type of germplasm bank

*Pilocarpus microphyllus* is an exceptional species because its seeds quickly lose viability under dry storage at low temperatures and exhibit high variability in germination percentages and seed viability (12–96%) among populations [29]. Therefore, preserving the species requires alternatives to seed banking, such as *in vitro* or field genebanks. Due to the limited availability of information and protocols for tissue culture and cryopreservation, we opted to preserve the genetic diversity of wild population in Carajás FLONA by establishing a living collection.

We opted for *inter situ* rather than *ex situ* living collections because our aim was to use the collection to enhance the understory of forests undergoing restauration in the Carajás region. Moreover, a semi-wild setting can increase plant reproduction and recruitment success [22]. The established of *inter situ* collections within degraded affordable landscapes allows to use large areas required to represent the genetic diversity and local adaptation of the species, as wild populations of *P. microphyllus* are genetically structured [32]. Thus planting individuals from each population source in isolated areas can maintain the adaptive genetic diversity and reduce the likelihood of exogamic depression [32,33]. Additionally, increasing the planted area could enhance the value of the collection, as more individuals planted are expected to generate more propagules for species recovery in the wild.

### Sampling design

Carajás National Forest (FLONA Carajás) is in the eastern Amazon, Brazil (6°03′32″ S, 50°10′23″ W), and covers an area of 3,927 km² [34]. The survey of *P. microphyllus* distribution and genetic sampling within FLONA Carajás is described in detail in [29,32]. Briefly, field expeditions conducted over six years, guided by species distribution models, assessed wild populations and produced a detailed occurrence map [29,32]. Subsequently, genetic diversity and population structure were evaluated using RAD sequencing (restriction site–associated DNA sequencing) to generate genome-wide SNP markers, based on young leaf samples collected from 20 individuals in each of 21 plant aggregates (420 individuals) across rainforest habitats and rocky outcrop edges within FLONA Carajás, following a stratified sampling design [29,32].

According to [29], *Pilocarpus microphyllus* exhibits significant genetic structure, comprising four geographically distinct population groups within the Carajás FLONA. Simulations estimating the minimum sampling effort required to capture genetic diversity indicated that collecting at least 40 individuals per genetic cluster (population), or 50 individuals randomly sampled across all clusters, would adequately represent the species’ genetic diversity in the region [29]. Based on these results, we designed an *inter situ* collection comprising at least 40 maternal lines per population (≥160 maternal lines in total), with each population established in a separate area. This design aims to preserve the long-term genetic diversity of wild populations, avoid gene flow among conspecifics from different populations, and produce seed material to support the persistence of these newly established populations as well as future conservation actions, including population reinforcement, reintroduction, and ecological restoration of degraded areas in Carajás.

### Plant germplasm acquisition: Harvest and post-harvest

#### Harvest and post-harvest propagating material.

Fruit collection was conducted between 2020 and 2023, rather than in a single year, to maximize genetic diversity captured [35]. Mature fruits were collected by the local CoEx-Carajás cooperative of collectors across the four populations of Carajás FLONA during the species peak fruiting period, from August to September [29]. Due to the high variation in seed germination and limited knowledge of seedling and sapling survival, up to 20% of each plant’s ripe reproductive output was collected to avoid over-collection [29,36]. Seeds were harvested at the individual level, with a minimum distance of 20 meters between sampled plants to increase the genetic diversity captured. Each maternal line was sampled only once over the years, with harvest efforts yearly adjusted according to sapling establishment success in the preceding year and current seed production.

**Fig 1:**
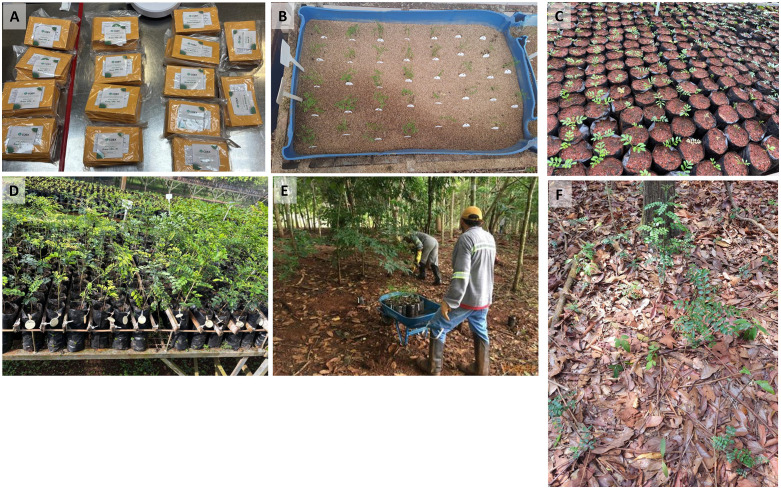
Overview of seed harvest, propagation, establishment, and monitoring of the jaborandi inter situ collection. Fruit harvest at the individual level, with each paper bag corresponding to one sampled maternal line **(A)**. Seed germination in vermiculite batches labeled with the maternal line code **(B)**. Seedling growth in the nursery prior to acclimatization (C) and during acclimatization **(D)**. Planting of jaborandi saplings in protected restoration areas within the Carajás National Forest (FLONA) **(E)**. Monitoring of plant survival and growth in the inter situ collection **(F)**.

All fruits collected from a given maternal plant (maternal line) were assigned a unique maternal code. Ripe fruits were placed in separate paper bags and labeled with this code, along with the collection date and geographic coordinates (Fig 1A). The bags were then transported to the Vale’s nursery facility at Serra dos Carajás. Within 30 days of harvesting, the seeds were manually extracted, and poorly developed or insect-infested seeds were removed. The extracted seeds were then stored for up to 60 days in a seed storage chamber at 20% relative humidity and a temperature of 5–10°C until they were propagated in the nursery.

### Establishment and management of *inter situ* collection

#### Propagation: seed germination and sapling production.

**Fig 2:**
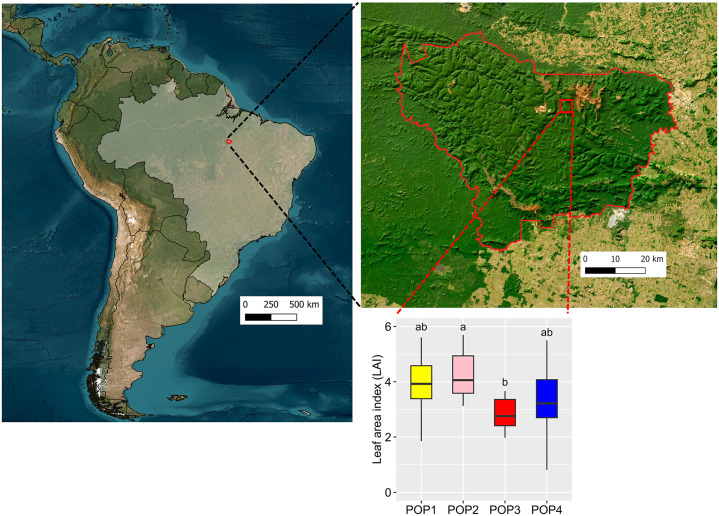
Location of the *Pilocarpus microphyllus* *inter situ* collection in the Carajás National Forest (Eastern Amazon, northern Brazil), on the left; leaf area index of the inter situ collection in four restoration areas, on the right. Each restoration area was planted with saplings derived from a unique wild population source. The map was drawn with QGIS software using the IBGE administrative boundaries shapefile (https://www.ibge.gov.br/geociencias/organizacao-do-territorio/malhas-territoriais.html), the MapBiomas land-cover data (https://plataforma.brasil.mapbiomas.org/). All data sources are public-domain and licensed under CC BY.

The jaborandi plants were propagated, cultivated and acclimated in the Vale nursery before being planted in the field. The Vale nursery, located in Carajás FLONA, closely mimics the climatic conditions of the species natural habitat. For propagation, seeds from each maternal line were sown in batches of vermiculite in a shaded greenhouse with 70% of light retention, and each batch was tagged with the maternal code (Fig 1B). The seedlings were kept in vermiculite until their first true leaves expanded. They were then transferred to individual 1 dm^3^ bags filled with growing substrate (Carajás forest topsoil and fertilizer), tagged with the maternal code, and moved to a greenhouse with 50% sunlight retention for acclimatization over five months (Fig 1B-1C). Acclimatization occurred under shaded conditions, as jaborandi is an understory plant, and the *inter situ* collection was established in areas undergoing rehabilitation with moderate canopy cover (leaf area index of 2–4, Fig 2). Plants that survived acclimatization were labeled with an accession number, and had their height, stem diameter, and number of leaves were recorded (Fig 1D).

### Establishment of *inter situ* collection

The living collections were established in four protected restoration areas within the Carajás FLONA, corresponding to former sand mines currently undergoing ecological restoration at different recovery stages [37]. All restoration areas are surrounded by submontane tropical rainforest and received a standard protocol for rehabilitation. A 30 cm layer of topsoil from nearby iron mining was applied to cover the mining substrate before planting native seedlings and seeds. Because the applied topsoil was acidic, sandy loam depleted in nutrients, soil amendments (pH correction and fertilization) were carried out prior to planting [37]. The jaborandi *inter situ* collections were placed in areas varying from 8 to 16 years-old of rehabilitation (Fig 1E), with indicators of convergence to nearby forest areas, such as vegetation structure and diversity [37]. These areas were chosen for their similarity to the ecological conditions of wild jaborandi populations, and recent leaf area index (LAI) measurements suggest for a well-developed forest canopy at all four sites (Fig 2). This convergence in the development of a forest understory would help to reduce plant losses due to a lack of adaptation, while supporting plant reproduction and maintaining local adaptations [8,33,38].

All saplings that survived acclimatization were transplanted to the field. Jaborandi saplings from distinct population sources were planted in separate areas, with each area located at least 1 km apart to minimize the risk of outbreeding depression, given the limited flight range of potential pollinators [32]. The saplings were planted during the rainy season in 30 × 30 × 30 cm holes, spaced 1 m apart within rows and 2 m between rows. Each planting hole was fertilized with 150 g of NPK (04 –14 –08) fertilizer. Each individual in the *inter situ* collection was georeferenced, mapped, and labeled with the planting date and an accession number.

### Management of *inter situ* collection

Ten months after planting, at the beginning of wet season, a topdressing fertilization of 200 g of NPK 20-00-20 was applied to each sapling. Periodic irrigation was carried out during the dry season, particularly from August to October. Plant survival, height, stem diameter, and number of leaves were recorded at the end of the dry season in September 2021, 2022, and 2023 (Fig 1F). Systematic monitoring of jaborandi propagation success and field establishment at an individual level provided feedback that was used to refine sampling strategies and guide subsequent seed harvesting efforts to achieve at least 40 maternal lines per population.

### Pre and pos-planting success

The ultimate goal of this *inter situ* collection is to establish four new self-sustaining jaborandi populations, each representing the genetic diversity of a distinct wild population within the Carajás FLONA. However, as individuals have not yet reached reproductive maturity, collection success was evaluated using short-term performance criteria [26,28]. We assessed four performance metrics annually: [1] the success of each propagation stage—germination, acclimatization, and field survival; [2] overall propagation success; [3] the number of plants surviving after one year in the field; and [4] the number of maternal lines per population source represented in the collection. Additionally, we determined the current stage of the collection based on the number of plants and maternal lines established as of 2023 (one- to three-year-old individuals).

The success of each propagation stage was used to identify developmental bottlenecks, defined as stages with success rates below 50%. Germination success was calculated as the mean proportion of seeds that germinated per maternal line; acclimation success as the mean proportion of germinated seedlings that survived acclimatization; and field survival as the mean proportion of acclimatized saplings that survived to become one-year-old field saplings per maternal line. Overall propagation success (henceforth propagation success) is an integrated measure across developmental transitions from seed to field-established saplings. Propagation success was calculated as the mean proportion of harvested seeds per maternal line that developed into one-year-old field saplings.

To examine inter- and intra-population variation in germination, we compared two models of seed germination probability for each harvest year: [1] a model including population as a fixed effect, and [2] a model including population as a fixed effect and maternal line as a random effect. Model 1 was fitted using a generalized linear model (GLM), whereas Model 2 was fitted using a generalized linear mixed model (GLMM). In both models, germination was coded as a binomial response variable (germinated vs. not germinated) and fitted using a binomial error distribution and logit link function. Model performance was compared using Akaike’s Information Criterion (AIC) and a likelihood ratio teste (χ²). All analyses were conducted using *lme4* and *emmeans* packages in R [39].

## Results

### Pre and pos-planting success

Seed harvest, propagation success, and bottleneck depended on both the year and the population source (Table 1). Consequently, the number of saplings planted (funder size) and established in the field per maternal line varied among populations and years. Seeds harvested in 2020 showed moderate propagation success for populations 1, 2, and 3, whereas population 4 exhibited very low success, constrained by acclimatization and sapling establishment in the field (Table 1). Although population 3 showed the greatest propagation success among the 2020 seeds, few maternal lines were sampled due to a low fruiting activity throughout that year (Table 1). Therefore, low fruiting activity was an important propagation bottleneck for this population. Unlike the 2020 harvest campaign, seeds harvested in 2021 from all populations showed a very low propagation success (Table 1). Seed germination significantly limited the propagation success for populations 1, 3, and 4, while sapling establishment posed a significant challenge for populations 2 and 4 (Table 1).

**Table 1:** Propagation success of seeds harvested in 2020 and 2021.

Seeds harvested in 2020							
Population	Maternal lines sampled	Harvested seeds	Germinated seeds	Acclimated saplings	Saplings in field	Established maternal lines	Propagation success from seed to 1year sapling
POP1	104	8.79 ± 6.09 (914)	0.71 ± 0.29 (673)	0.67 ± 0.99 (440)	0.99 ± 0.06 (429)	88	0.477
POP2	50	11.22 ± 5.31 (561)	0.77 ± 0.25 (445)	0.57 ± 0.41 (248)	0.96 ± 0.16 (237)	34	0.398
POP3	**8**	29.25 ± 35.7 (234)	0.73 ± 0.32 (183)	0.72 ± 0.32 (155)	1 ± < 0.01 (155)	7	0.531
POP4	39	21.95 ± 19.64 (856)	0.68 ± 0.25 (614)	**0.43 ± 0.42 (279)**	**0.2 ± 0.29 (55)**	26	0.055
Seeds harvested in 2021							
POP1	129	13.01 ± 5.5 (1678)	**0.21 ± 0.38 (448)**	0.75 ± 0.18 (335)	0.51 ± 0.32 (163)	16	0.080
POP2	100	28.76 ± 14.22 (2876)	0.53 ± 0.33 (1760)	0.98 ± 0.02 (1724)	**0.23 ± 0.26 (367)**	41	0.121
POP3	151	9.7 ± 5.47 (1465)	**0.01 ± 0.07 (11)**	0.91 ± NA (10)	0.81 ± NA (8)	1	0.007
POP4	160	38.63 ± 10.79 (6736)	**0.37 ± 0.38 (2754)**	0.91 ± 0.232 (2500)	**0.18 ± 0.25 (518)**	51	0.061

Maternal lines sampled (number of maternal lines sampled); harvested seeds (mean harvested seeds by maternal line ± standard deviation, and total harvested seeds by population); germinated seed (mean proportion of germinated seeds by maternal line ± sd, and total germinated seeds by population); acclimated sampling (mean proportion of germinated seeds that survived to acclimatization by maternal line ± sd, and total acclimatized saplings by population; saplings in field (mean proportion of one-year saplings that survived in field by maternal line with regard to acclimatized saplings ± sd, and total 1-year saplings by population); established maternal lines (number of maternal lines represented by 1-year saplings). Success proportion from seed to 1year sapling (the mean proportion of seeds that become established one-year saplings in the field by maternal line) Bottleneck propagation stage in bold, i.e., stage with success lower than 50%.

The number of plants surviving the first year in the field increased significantly with the number of individuals planted (i.e., founder size; Pearson correlation: r = 0.73, t = 2.61, df = 6, p = 0.04). Founder size and first-year survival were generally high (>150 individuals) across populations in both harvest years, except for population 3 in the 2021 harvest (for both metrics) and population 4 in 2020, which showed a reduced number of first-year survivors (Table 1). Although the proportion of first-year survival in population 4 was low (ca. 20%) in both harvest years, the absolute number of one-year-old saplings established in 2021 remained high (518 individuals) due to the large founder size (2,500 saplings planted).

The 2022 harvest campaign focused on populations 2, 3, and 4 because population 1 already had a large number of established maternal lines (104) and more than 500 plants established in the field (Table 1). Despite harvesting more than 13,000 seeds from 294 maternal lines, the nursery was affected by a rat infestation, resulting in the loss of 66% of maternal lines due to seed and seedling predation. Nevertheless, 302 plants representing 57 maternal lines from population 2 survived the first year in the field; 115 plants representing 9 maternal lines from population 3; and 655 plants representing 33 maternal lines from population 4.

**Fig 3:**
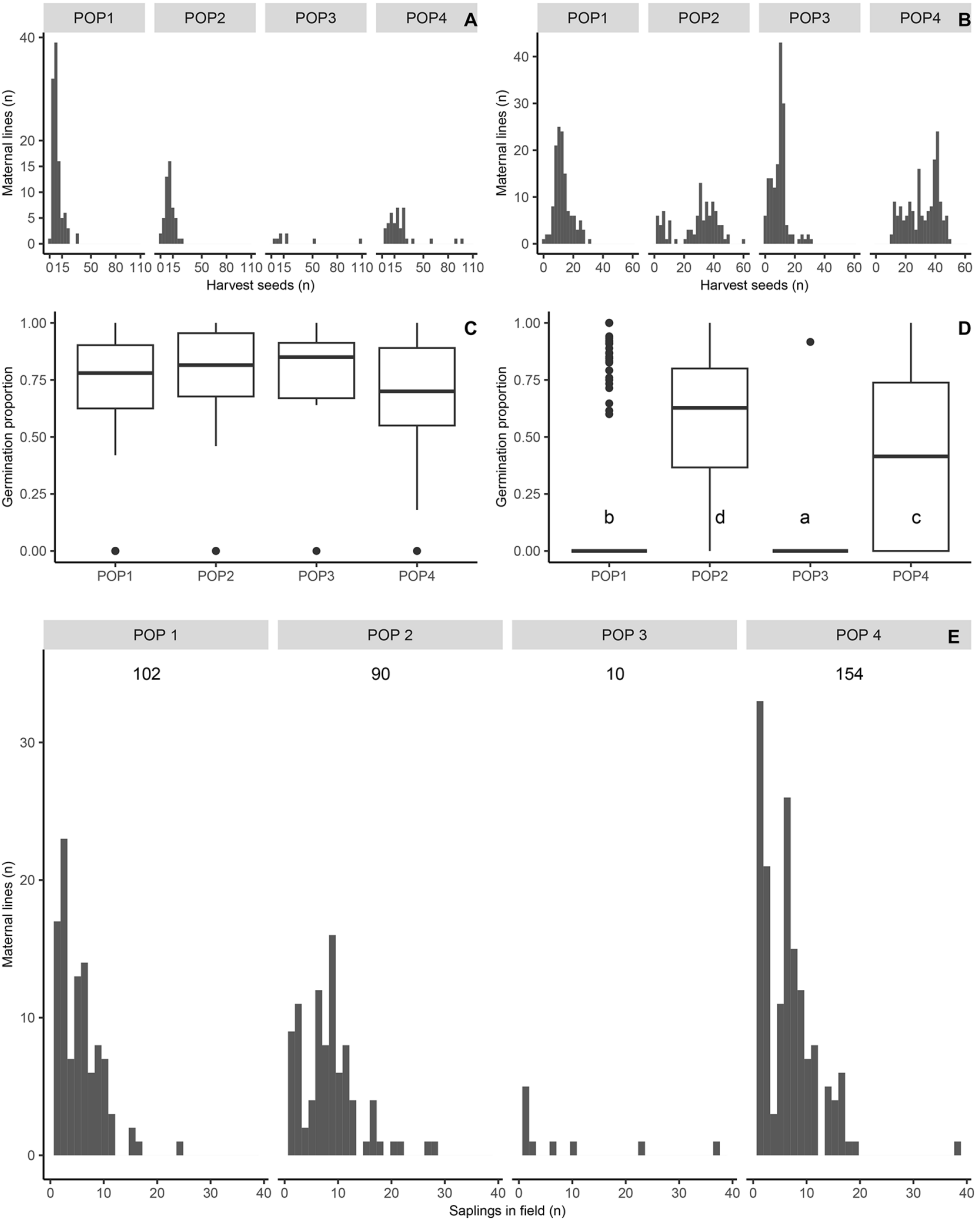
Harvest effort by maternal line of *Pilocarpus microphyllus* in 2020 (A) and 2021 (B). Inter and intra-population variation in seed germination in 2020 (C) and 2021 (D). Distribution of saplings with ≥ one-year old by maternal line in each population in July 2023 (E). Number of maternal lines established in field showed in the top of panel E. Different letters indicate significant differences in germination proportion (C and D panels) according to the GLMM with binomial error distribution: Germination (1,0) ~ Population + (1 | Maternal line).

Seed germination varied widely both among and within populations, as models accounting for maternal line variation within populations provided a better fit than models considering population differences alone (S1 Table). Seeds harvested in 2020 showed high germination percentages across populations (median >70%; Fig 3C), although a moderate proportion of germination variation (24%) was explained by intrapopulation differences (Table 2). In contrast, germination in the 2021 harvest differed significantly among populations (Fig 3D), and intrapopulation variation accounted for a large proportion of the total germination variance (87%; Table 3).

**Table 2:** Effect of population source and maternal line source on germination proportion of jaborandi seed harvest in 2020 from the Carajás FLONA.

Coefficients	Estimate	Standard error	z value	p-value
Intercept	1.19	0.14	8.68	**< 0.001**
Population 2	0.35	0.23	1.48	0.14
Population 3	0.04	0.46	0.08	0.94
Population 4	−0.28	0.23	−1.21	0.22
Groups	Number groups	Stand. Dev.	ICC	
Maternal line	201	1.01	0.24	

Results for the logistic regression to evaluate the intra and interpopulation effect on seed germination. ICC = Intraclass Correlation Coefficient indicates that 24% of variance in germination proportion was explained by differences within the population. P-value indicates that seed germination did not differ between population.

**Table 3:** Effect of population origin and maternal line source on germination percentage of jaborandi seeds harvest in 2021 from the Carajás FLONA.

Coefficients	Estimate	Standard error	z value	p-value
Intercept	−5.84	0.57	−10.27	**<0.001**
Population 2	5.60	0.73	7.70	**<0.001**
Population 3	−4.44	1.36	−3.26	**0.001**
Population 4	2.96	0.63	4.71	**<0.001**
Groups	Number groups	Stand. Dev.	ICC	
Maternal line	536	4.65	0.87	

Results for the logistic regression to evaluate the intra and interpopulation effect on seed germination. ICC = Intraclass Correlation Coefficient indicates that 87% of variance in germination proportion is explained by differences within the population. P-value indicates that seed germination differs between population.

### Current stage of the jaborandi *inter situ* collection

The number of maternal lines represented by one- to three-year-old saplings in 2023 was more than twice the minimum sampling effort required to capture genetic diversity (40 maternal lines per population) for populations 1, 2, and 4 (Fig 3E). This threshold was estimated through simulations based on the species’ genetic structure [29]. Furthermore, most maternal lines from these populations are represented by more than three replicates and by a large number of surviving plants (552, 755, and 1,017 individuals for populations 1, 2, and 4, respectively). In contrast, population 3 is underrepresented in the collection, with only 10 maternal lines established, half of which are represented by a single replicate and relatively few surviving individuals (85 plants) (Fig 3E).

## Discussion

### Short-term performance of the *inter situ* collection

The jaborandi *inter situ* collection exhibited relatively high short-term success compared with global conservation translocation case studies that used juveniles or seedlings as founders [14,28,40], although *inter situ* success rates remain poorly documented. First-year survival in the jaborandi collection ranged from 44 to 78%, representing the mean survival across populations in 2020 and 2021, and was comparable to mean global estimates (65%) [27], with more than 50% survival reported in 72% of Australian conservation translocations [28], approximately 60% survival in Mediterranean woody species [40], and 47–96% survival reported for Atlantic Forest species, with most species (4 of 6) showing more than 80% survival [41]. However, survival percentage of jaborandi *inter situ* collection ranged from 20 to 100% depending on population and year, with population 4 showing consistently low survival (ca. 20%) in both 2020 and 2021, and reduced survival also observed in population 2 in 2021.

Despite low-survival percentages observed in population 4, the number of plants surviving the first year remained comparatively high, ranging from 55 to 429 individuals in 2020 and from 8 to 518 in 2021 across populations, with more than 150 plants surviving after the first planting year in three populations (75%) in both years. Similar establishment outcomes have been reported for conservation translocations in the Atlantic Forest of Brazil, where first-year survival ranged from 137 to 607 individuals, with more than 225 surviving plants in four of six study species [37]. At broader spatial scales, conservation translocations typically report substantially lower numbers of established individuals, with 62% of projects showing fewer than 50 surviving plants and only 13% exceeding this threshold [28]. Australian rainforest translocations show even lower establishment outcomes, with 80% of projects reporting fewer than 50 surviving individuals [28].

Our results showed a strong relationship between founder size and the number of surviving plants, explaining the high number of established individuals even under low survival percentages observed in population 4. Large founder sizes were used in most populations (155–2500 saplings across years), except for population 3 in 2021 (10 saplings), where founder size was constrained by low seed germination. Similar use of large founder numbers has been reported in conservation translocations in the Atlantic forest, where 221–750 saplings were introduced [41]. In contrast, many conservation translocations worldwide rely on substantially smaller founder sizes due to limited propagule availability [28,42]. For example, approximately 45% of Australian translocations use fewer than 50 founder propagules and only 16% exceed 250 individuals, with Australian rainforest translocations showing even lower founder sizes, where 75% of projects use fewer than 50 propagules [28]. Founder size is widely recognized as a reliable predictor of translocation success, as survival and recruitment probability generally increase with the number of introduced individuals [27,28,40].

Taken together, these demographic outcomes indicate that the *inter situ* collection has achieved substantial short-term establishment success for three of the four new jaborandi populations, as they exhibit a high number of established plants and adequate genetic representation of wild populations [26,28]. For jaborandi populations 1, 2, and 4, the large number of surviving one- to three-year-old individuals (>500 plants per population) is likely sufficient to promote demographic stability, increase the probability of plant recruitment, and the long-term population persistence [28]. Moreover, these populations are well represented genetically in the *inter situ* collection, as the number of established maternal lines exceeds by more than twofold the minimum sampling effort required to capture the genetic diversity of wild populations [32]. In contrast, population 3 remains genetically underrepresented in the collection, with only 25% of the targeted maternal lines established. In addition, the relatively low number of surviving individuals (85) may limit its future viability [28]. Additional seed-harvesting campaigns should prioritize population 3 to increase its genetic representation and strengthen population establishment. At the same time, continued sampling of all populations will be important to support understory enrichment at the *inter situ* collection sites, thereby enhancing propagule production and reinforcing the long-term conservation value of the collection.

### Pre and pos-planting bottlenecks

Founder size, first-year survival, and site suitability are among the strongest predictors of *inter situ* collection success [35–37]. Likewise, seedling and juvenile establishment are widely recognized as major bottlenecks limiting the formation of viable populations and overall translocation success [36,38]. Consequently, *inter situ* collection success has been primarily evaluated through post-planting demographic performance. However, our results show that both pre- and post-planting processes varied among populations and years, affecting population size and genetic diversity through differences in founder size and in the number of established maternal lines.

Pre-planting processes strongly and consistently influenced founder size and maternal line representation in population 3. For this population, seed production represented a major bottleneck in both harvest years due to few fruiting individuals in 2020 and low seed production per individual in 2021. In addition, extremely low seed germination (1%) in 2021 substantially reduced founder size, from more than 1,400 harvested seeds to only 11 seedlings and resulted in the loss of several maternal lines prior to planting (151 sampled to a single established maternal line). Conservation translocations in the Atlantic Forest similarly show that finding enough reproductive individuals and obtaining viable propagules can constrain translocation success when planting material is collected directly from wild populations [38]. Moreover, low germination can further constrain seedling production in conservation programs, reducing the efficiency of propagule production required for conservation translocations and restoration efforts [43]. Such low germination may arise from seed dormancy or reduced seed viability, limiting seedling availability even under controlled *ex situ* propagation conditions [43]. Together, these results indicate that bottlenecks occurring before planting—either during propagule collection in wild populations or during *ex situ* propagation—can substantially reduce founder size and genetic representation, ultimately influencing translocation outcomes prior to field establishment.

The reduced reproductive output and low germination observed in population 3 may arise from multiple, non-mutually exclusive processes, genetic limitations are unlikely to explain these patterns, as this population exhibited high heterozygosity, negative inbreeding coefficients, and the largest effective population size among populations, indicating no evidence of genetic erosion or inbreeding depression [29]. Instead, ecological or reproductive constraints may underlie the observed pre-planting bottleneck. Jaborandi populations occurring in extractive-use areas may experience recurrent leaf harvesting, which can reduce plant vigor and carbon allocation to reproduction, ultimately limiting flower and fruit production.

Environmental conditions during seed development and intrinsic seed traits may further contribute to reduced germination [43,44]. Low germination can result from factors such as seed dormancy or reduced seed viability, both of which may limit seedling production even under controlled *ex situ* propagation. However, whether *P. microphyllus* seeds exhibit dormancy, and how dormancy or seed viability vary within and among populations, remains unknown. Variation in these seed traits may therefore further influence germination success and propagule availability, reinforcing pre-planting bottlenecks that ultimately affect conservation translocation outcomes.

Post-planting performance represented an important bottleneck for population 4, as field establishment was particularly low, with up to 80% of saplings lost after transfer. The reduced establishment observed in this population is unlikely to result from low genetic diversity, as it exhibited levels of heterozygosity and nucleotide diversity comparable to those of successfully established populations, as well as a moderate effective population size [32]. However, the slightly positive inbreeding coefficient—suggesting a mild excess of homozygosity—together with historical population contractions followed by limited expansion may have resulted in the loss of locally adaptive variants or reduced ecological plasticity, making individuals less suited to establishment under restoration conditions [45].

Alternatively, low establishment proportion may be related to inadequate site or microhabitat selection at the recipient location [27,28]. Leaf area index (LAI) at the population 4 site did not differ significantly from that of the other *inter situ* locations, indicating that variation in vegetation structure, canopy density, and understory light availability is unlikely to explain the observed differences in establishment among populations. Moreover, all jaborandi’ *inter situ* collection sites show convergence with nearby forest areas in terms of vegetation structure and diversity [33]. Therefore, microhabitat conditions other than vegetation structure or plant diversity may influence sapling establishment. Further research assessing soil physicochemical properties and soil microbial communities in the wild population 4 site and in the *inter situ* collections may help elucidate the primary drivers of sapling survival, which is crucial for optimizing *inter situ* conservation strategies and informing jaborandi restoration efforts.

### Learnings and next steps

Our case study highlights the importance of accounting for plant loss across different life stages—from seed germination to juvenile field survival—to identify bottlenecks and guide management actions that maximize founder production and survival. The substantial interannual variation in germination percentage observed within and among populations suggests that *P. microphyllus* may exhibit seed dormancy or that seed viability is highly variable. Therefore, studies assessing whether jaborandi seeds exhibit dormancy and identifying the environmental cues required to break dormancy could help increase seedling production. In contrast, the low percentage of juvenile establishment observed in population 4 suggests limited microsite suitability for plant establishment. Consequently, experimental approaches evaluating how variation in soil and microsite conditions influences juvenile survival could help optimize the establishment phase [27,46]. Increasing founder production and post-planting survival is critical for the success of *inter situ* collections, as founder size is a key determinant of establishment success, and relatively large founder populations (ca. 350–500 individuals) are often required to achieve self-sustaining populations [27,28].

Systematic monitoring of jaborandi propagation success and field establishment at the individual level enabled us to identify propagation bottlenecks and provided feedback to refine sampling strategies and inform seed-harvesting efforts aimed at improving maternal line establishment. Two approaches can be used to estimate the appropriate seed harvest effort for future campaigns: (1) examining the relationship between harvest effort and the number of maternal lines established across different levels of propagation success, or (2) dividing the target establishment probability (e.g., > 75%) by the mean proportion of seeds from each maternal line that become established one-year-old saplings in the field.

When propagation success was moderate (≥40%), increasing the number of maternal lines sampled while reducing the harvest effort to 8–12 seeds per individual resulted in a higher number of maternal lines successfully established in the field (e.g., populations 1 and 2 vs. population 3 in 2020; Table 1). Conversely, when propagation success was low—as observed for seeds harvested from all populations in 2021 and from population 4 in 2020—a higher harvest effort per maternal line (>28 seeds) was required to successfully establish a large number of maternal lines in the field (populations 2 and 4 vs. populations 1 and 3; Table 1). Complementarily, the second approach indicates that the mean harvest effort required to achieve a > 75% establishment probability—averaging the worst-case propagation success across all four populations—is 33 seeds (ranging from 6 to 107 seeds in the most extreme case). Based on these integrated results, we recommend harvesting 20–30 seeds per maternal line, from as many maternal lines as possible in each harvesting campaign. These recommendations should follow the guideline of collecting no more than 20% of the seeds produced per individual to minimize impacts on natural populations while maximizing the number of maternal lines successfully established in the field.

The significant variation in germination percentage observed within and among populations further supports this sampling strategy, as a large proportion of the variation in germination was attributed to differences among maternal lines within populations. Consequently, sampling as many maternal lines as possible increases the likelihood of collecting seeds from individuals with high germination capacity within each population. At the same time, increasing the harvest effort to 20–30 seeds per maternal line may improve the probability of establishing at least one seedling from maternal lines with lower germination capacity.

Early collection performance indicates that the jaborandi *inter situ* collection has been successfully established, with high numbers of surviving plants and broad genetic representation of wild populations. However, early plant performance does not necessarily predict long-term population persistence [42], and the ultimate success of conservation translocations can only be evaluated once individuals reach reproductive maturity and natural recruitment occurs. This highlights the importance of long-term monitoring to assess whether the *inter situ* populations become demographically self-sustaining. In addition, demographic studies of the wild populations are needed to provide baseline parameters for evaluating the performance of the *inter situ* collections. Monitoring plant survival, growth, reproduction, and recruitment in both wild and planted populations will enable the development of population viability models, which can guide adaptive management and improve future conservation actions.

## Conclusions

Our study provides strong evidence that *inter situ* collection can effectively capture the genetic diversity of jaborandi within the Carajás National Forest. Current short-term success criteria indicate that this collection is in the way to the long-term success. Furthermore, establishing jaborandi in restoration areas within the Carajás region paves the way for ecological restoration using threatened species, though further research into sapling establishment remains essential to fully optimize these efforts.

A key finding of our work is that the probability of a harvested seed becoming an established one-year-old sapling varies significantly—from 7% to 53%—depending on the population and harvest year. As a substantial portion of this variation is attributed to within-population differences, we underscore the critical importance of systematically monitoring propagation and field establishment at the individual level. Such monitoring is vital to refine sampling strategies over time, optimize harvesting efforts, and provide nurseries and restoration practitioners with the data needed to determine the resources required for production.

Ultimately, we hope that our case study using a threatened exceptional species will encourage the adoption of *inter situ* collections as a strategic approach to integrate *ex situ* and *in situ* conservation. This is especially relevant for species in moist tropical forests, where traditional seed banking is often not feasible and where *in situ* measures alone may be insufficient to prevent extinction [12].

## Supporting information

S1 Table Competing general linear model and general linear mix models tested to evaluated the effect of individual variation on seed germination percentage of jaborandi populations from the Carajás FLONA harvest in 2020 and 2021. Pop. = population; d.f. = degrees of freedom; Best models according to the lowest AIC models in bold; p-value of X^2^ test in bold indicate that models significative differ in the capacity to explain the variation of germination percentage.
(DOCX)

S2 Table Raw data of seed harvest and propagation success of jaborandi seed harvest in 2020 and 2021 from the Carajás FLONA. (XLSX)

S1 File Supplementary guidelines. (DOCX)

## References

[1] Brinck K, Fischer R, Groeneveld J, Lehmann S, Dantas De Paula M, Pütz S, et al. High resolution analysis of tropical forest fragmentation and its impact on the global carbon cycle. Nat Commun. 2017;8:14855. doi: 10.1038/ncomms14855 28303883 PMC5357863

[2] Hansen MC, Wang L, Song XP, Tyukavina A, Turubanova S, Potapov PV. The fate of tropical forest fragments. Sci Adv. 2020;6(11):eaax8574. doi: 10.1126/sciadv.aax8574 PMC7065873 32195340

[3] Raisg Ra de ISG. Amazônia sob pressão. São Paulo: RAISG - Rede Amazônica de Informação Socioambiental Georreferenciada; 2020.

[4] Intergovernmental Panel On Climate Change. Climate change and land: IPCC special report on climate change, desertification, land degradation, sustainable land management, food security, and greenhouse gas fluxes in terrestrial ecosystems. 1st ed. Cambridge University Press; 2022. doi: 10.1017/9781009157988

[5] Convention on Biological Diversity. The global strategy for plant conservation: 2011-2020. Richmond, UK: Botanic Gardens Conservation International; 2012.

[6] Volis S. Conservation-oriented restoration - a two for one method to restore both threatened species and their habitats. Plant Divers. 2019;41(2):50–8. doi: 10.1016/j.pld.2019.01.002 31193129 PMC6520488

[7] Center for Plant Conservation. CPC Best Plant Conservation Practices to Support Species Survival in the Wild. Escondido, CA: Center for Plant Conservation; 2019.

[8] FAO. Genebank standards for plant genetic resources for food and agriculture. Roma, Italy: FAO; 2014.

[9] Martyn Yenson AJ, Sommerville KD, Guja LK, Merritt DJ, Dalziell EL, Auld TD, et al. Ex situ germplasm collections of exceptional species are a vital part of the conservation of Australia’s national plant treasures. Plants People Planet. 2023;6(1):44–66. doi: 10.1002/ppp3.10421

[10] Potter KM, Jetton RM, Bower A, Jacobs DF, Man G, Hipkins VD, et al. Banking on the future: progress, challenges and opportunities for the genetic conservation of forest trees. New Forests. 2017;48(2):153–80. doi: 10.1007/s11056-017-9582-8

[11] Pence VC, Meyer A, Linsky J, Gratzfeld J, Pritchard HW, Westwood M, et al. Defining exceptional species—A conceptual framework to expand and advance ex situ conservation of plant diversity beyond conventional seed banking. Biol Conserv. 2022;266:109440. doi: 10.1016/j.biocon.2021.109440

[12] Wyse SV, Dickie JB, Willis KJ. Seed banking not an option for many threatened plants. Nat Plants. 2018;4(11):848–50. doi: 10.1038/s41477-018-0298-3 30390079

[13] Pence VC, Bruns EB, Meyer A, Pritchard HW, Westwood M, Linsky J. Gap analysis of exceptional species—Using a global list of exceptional plants to expand strategic ex situ conservation action beyond conventional seed banking. Biol Conserv. 2022;266:109439. doi: 10.1016/j.biocon.2021.109439

[14] Yenson M, Amelia J, Offord CA, Meagher PF, Auld TD, Bush D. Plant germplasm conservation in Australia. 3rd ed. Canberra: Australian Network for Plant Conservation; 2021.

[15] Maxted N, B J, Magos S, Kell. Resource book for the preparation of national plans for conservation of crop wild relatives and landraces. Birmingham, UK: University of Birmingham; 2013.

[16] Colville L, Pritchard HW. Seed life span and food security. New Phytol. 2019;224(2):557–62. doi: 10.1111/nph.16006 31225902

[17] Westwood M, Cavender N, Meyer A, Smith P. Botanic garden solutions to the plant extinction crisis. Plants People Planet. 2020;3(1):22–32. doi: 10.1002/ppp3.10134

[18] Diaz-Martin Z, Fant J, Havens K, Cinea W, Tucker Lima JM, Griffith MP. Current management practices do not adequately safeguard endangered plant species in conservation collections. Biol Conserv. 2023;280:109955. doi: 10.1016/j.biocon.2023.109955

[19] Cochrane JA, Barrett S, Monks L, Dillon R. Partnering conservation actions. Inter situ solutions to recover threatened species in South West Western Australia. Kew Bull. 2010;65(4):655–62. doi: 10.1007/s12225-010-9233-0

[20] Coates DJ, Byrne M, Moritz C. Genetic diversity and conservation units: dealing with the species-population continuum in the age of genomics. Front Ecol Evol. 2018;6:165. doi: 10.3389/fevo.2018.00165

[21] Quiroga MP, Castello L, Quipildor V, Premoli AC. Biogeographically significant units in conservation: a new integrative concept for conserving ecological and evolutionary processes. Environ Conserv. 2019;46(4):293–301. doi: 10.1017/S0376892919000286

[22] Burney DA, Burney LP. Paleoecology and “inter-situ” restoration on Kaua’i, Hawai’i. Front Ecol Environ. 2007;5(9):483–90. doi: 10.1890/070051

[23] Volis S. Complementarities of two existing intermediate conservation approaches. Plant Divers. 2017;39(6):379–82. doi: 10.1016/j.pld.2017.10.005 30159532 PMC6112325

[24] Commander LE, Coates DJ, Broadhurst L, Offord CA, Makinson RO, Matthes M. Guidelines for the translocation of threatened plants in Australia. 3rd ed. Canberra: Australian Network for Plant Conservation; 2018.

[25] Kueffer C, Kaiser-Bunbury CN. Reconciling conflicting perspectives for biodiversity conservation in the Anthropocene. Front Ecol Environ. 2014;12(2):131–7. doi: 10.1890/120201

[26] Monks L, Barrett S, Beecham B, Byrne M, Chant A, Coates D, et al. Recovery of threatened plant species and their habitats in the biodiversity hotspot of the Southwest Australian Floristic Region. Plant Divers. 2018;41(2):59–74. doi: 10.1016/j.pld.2018.09.006 31193161 PMC6520493

[27] Maschinski J, Coates D, Monks L, Dillon R, Barrett S, Possley J. Rare and threatened plant conservation translocations: Lessons learned and future directions. In: Florentine S, Gibson-Roy P, Dixon KW, Broadhurst L, editors. Ecological restoration. Cham: Springer International Publishing; 2023. 287–322. doi: 10.1007/978-3-031-25412-3_8

[28] Silcock JL, Simmons CL, Monks L, Dillon R, Reiter N, Jusaitis M. Threatened plant translocation in Australia: a review. Biol Conserv. 2019;236:211–22. doi: 10.1016/j.biocon.2019.05.002

[29] Caldeira CF, Giannini TC, Ramos SJ, Vasconcelos S, Mitre SK, Pires JPDA. Sustainability of jaborandi in the eastern Brazilian amazon. Perspect Ecol Conserv. 2017;15(3):161–71. doi: 10.1016/j.pecon.2017.08.002

[30] Fernandez E, Gomes M, Ferreira GC, Marinho IA. Pilocarpus microphyllus Stapf ex Wardlew. (Rutaceae) in Lista Vermelha da Flora Brasileir [Internet]. Rio de Janeiro: Centro Nacional de Conservação da Flora/ Instituto de Pesquisas Jardim Botânico do Rio de Janeiro; 2021. https://cncflora.jbrj.gov.br/ficha/882

[31] Boulton CA, Lenton TM, Boers N. Pronounced loss of Amazon rainforest resilience since the early 2000s. Nat Clim Chang. 2022;12(3):271–8. doi: 10.1038/s41558-022-01287-8

[32] Monteiro WP, Dalapicolla J, Carvalho CS, Costa Veiga J, Vasconcelos S, Ramos SJ. Genetic diversity and structure of an endangered medicinal plant species (Pilocarpus microphyllus) in eastern Amazon: implications for conservation. Conserv Genet. 2022;23(4):745–58. doi: 10.1007/s10592-022-01454-6

[33] Volis S, Blecher M. Quasi in situ: a bridge between ex situ and in situ conservation of plants. Biodivers Conserv. 2010;19(9):2441–54. doi: 10.1007/s10531-010-9849-2

[34] Plano de manejo da floresta nacional de Carajás: Volume I – diagnóstico. Brasilia: ICMBio; 2016.

[35] Griffith MP, Clase T, Toribio P, Piñeyro YE, Jimenez F, Gratacos X, et al. Can a botanic garden metacollection better conserve wild plant diversity? A case study comparing pooled collections with an ideal sampling model. Int J Plant Sci. 2020;181(5):485–96. doi: 10.1086/707729

[36] Di Sacco A, Way M, León Lobos P, Suárez Ballesteros CI, Díaz Rodriguez JV. Manual de recolección, procesamiento y conservación de semillas de plantas silvestres. Royal Botanic Gardens, Kew e Instituto de Investigación de Recursos Biológicos Alexander von Humboldt; 2020.

[37] Gastauer M, Caldeira CF, Ramos SJ, Silva DF, Siqueira JO. Active rehabilitation of Amazonian sand mines converges soils, plant communities and environmental status to their predisturbance levels. Land Degrad Dev. 2020;31(5):607–18. doi: 10.1002/ldr.3475

[38] Zinnen J, Broadhurst LM, Gibson-Roy P, Jones TA, Matthews JW. Seed production areas are crucial to conservation outcomes: benefits and risks of an emerging restoration tool. Biodivers Conserv. 2021;30(5):1233–56. doi: 10.1007/s10531-021-02149-z

[39] R Core Team. R: the R project for statistical computing. Accessed 2024 October 20. https://www.r-project.org/

[40] Fenu G, Bacchetta G, Christodoulou CS, Cogoni D, Fournaraki C, Gian Pietro G del G, et al. A common approach to the conservation of threatened island vascular plants: first results in the mediterranean basin. Diversity. 2020;12(4):157. doi: 10.3390/d12040157

[41] Soorae PS. Global conservation translocation perspectives: 2021: Case studies from around the globe. IUCN SSC Conservation Translocation Specialist Group; 2021.

[42] Dalrymple SE, Banks E, Stewart GB, Pullin AS. A meta-analysis of threatened plant reintroductions from across the globe. Plant reintroduction in a changing climate. Island Press/Center for Resource Economics; 2012. 31–50. doi: 10.5822/978-1-61091-183-2_3

[43] Kildisheva OA, Dixon KW, Silveira FAO, Chapman T, Di Sacco A, Mondoni A. Dormancy and germination: making every seed count in restoration. Restorat Ecol. 2020;28(S3). doi: 10.1111/rec.13140

[44] Baskin CC, Baskin JM. Seeds: ecology, biogeography, and evolution of dormancy and germination. 2nd ed. San Diego, CA: Elsevier/AP. 2014.

[45] Dillon R, Coates D, Standish R, Monks L, Waycott M. Aust J Bot. 2023;71(2):79–92. doi: 10.1071/BT22071

[46] Dillon R, Monks L, Coates D. Establishment success and persistence of threatened plant translocations in south west Western Australia: an experimental approach. Aust J Bot. 2018;66(4):338–46. doi: 10.1071/BT17187

