# Supplementary material for: Inter situ collections as a strategy to conserve an exceptional plant species from the Amazon rainforest
Source: PLoS One. 2026 Jun 3;21(6):e0349107. doi: 10.1371/journal.pone.0349107 (PMC13232789; doi:10.1371/journal.pone.0349107)
Supplement: S1 Table — Pop. = population; d.f. = degrees of freedom; Best models according to the lowest AIC models in bold; p-value of X2 test in bold indicate that models significative differ in the capacity to explain the variation of germination percentage. (DOCX) [file pone.0349107.s001.docx]

S1 Table. Competing general linear model and general linear mix models tested to evaluated the effect of individual variation on seed germination percentage of jaborandi populations from the Carajás FLONA harvest in 2020 and 2021.

| Seed harvest in 2020 | | | | |
| --- | --- | --- | --- | --- |
| Model | d.f. | AIC | Residual dev. | p-value |
| Germination ~ Pop. | 4 | 1034.38 | 659.66 |  |
| Germination ~ Pop. + (1\|maternal line) | 5 | **842.98** | 647.01 | **0.006** |
| Seed harvest in 2021 | | | | |
| Model | d.f. | AIC | Residual dev. | p-value |
| Germination ~ Pop. | 4 | 6944.04 | 8329.5 |  |
| Germination ~ Pop. + (1\|maternal line) | 5 | **1980.93** | 6317.7 | **<0.001** |

Pop. = population; d.f.= degrees of freedom; best models according to the lowest AIC models in bold; p-value of X^2^ test in bold indicate that models significative differ in the capacity to explain the variation of germination percentage.
