# Supplementary material for: Inter situ collections as a strategy to conserve an exceptional plant species from the Amazon rainforest
Source: PLoS One. 2026 Jun 3;21(6):e0349107. doi: 10.1371/journal.pone.0349107 (PMC13232789; doi:10.1371/journal.pone.0349107)
Supplement: S1 File — (DOCX) [file pone.0349107.s003.docx]

**Guidelines to preserve plant species through *inter situ* collections**

**Preparations before collecting propagation materials**

**Assess the suitability of inter situ conservation**

The first step in establishing an *inter situ* collection is to confirm whether this approach is appropriate for the target species, based on its biological characteristics and conservation goals. *Inter situ* collections are particularly suitable for species that cannot be efficiently conserved through seed banking and exhibit geographically structured populations, as they require isolated living collections to maintain adaptive genetic diversity or when the collections also aim to support restoration or reintroduction programs (1,2). Although most seed plants can be preserved in seed banks, approximately 20% of plant species—including non-seed plants, species with desiccation-sensitive seeds, and species with low seed viability or irregular reproductive cycles—cannot be conserved using this approach (2–4). As alternatives to *inter situ* collections, desiccation-sensitive seeds may be preserved through cryopreservation when embryos tolerate desiccation or when plants produce dormant buds; otherwise, long-term conservation relies on tissue culture or field genebanks (5,6).

**Sampling design**

Once the type of bank has been determined, the next step is to decide how many living plants to include in the collection and where to source them in order to preserve genetic diversity and evolutionary potential. This involves determining the number of individuals and populations to sample to include 95% of common alleles (7–9). High genetic diversity in conservation collections is crucial for species survival in the wild, as it underpins evolutionary change and adaptation to climate change, habitat, and biotic interactions (10,11).

The required number of individuals and populations depends on the breeding systems of the species in question, their modes of pollination and seed dispersal, and their demographic processes (12). This can be determined using molecular genetic data indicating genetic diversity and population structure (13) or demographic data, such as population numbers, sizes, and migration rates (14). Additionally, plant loss across different life stages must be accounted for in the sampling design (14). Seed viability, germination rates, and seedling and sapling mortality can inform the quantity of seeds to collect per plant (15). In the absence of genetic or demographic data, harvesting propagation material from approximately 200-300 individuals across the species' range may suffice to conserve 95% of all alleles (14). However, most germplasm collection manuals recommend sampling 30-200 individuals from at least five populations across the species' ecological and geographical range, typically 6-40 individuals per population (7,9). Detailed guidance on sampling design is available in (14,16–20), while the main steps for establishing an inter situ collection are summarized in Figure 1.


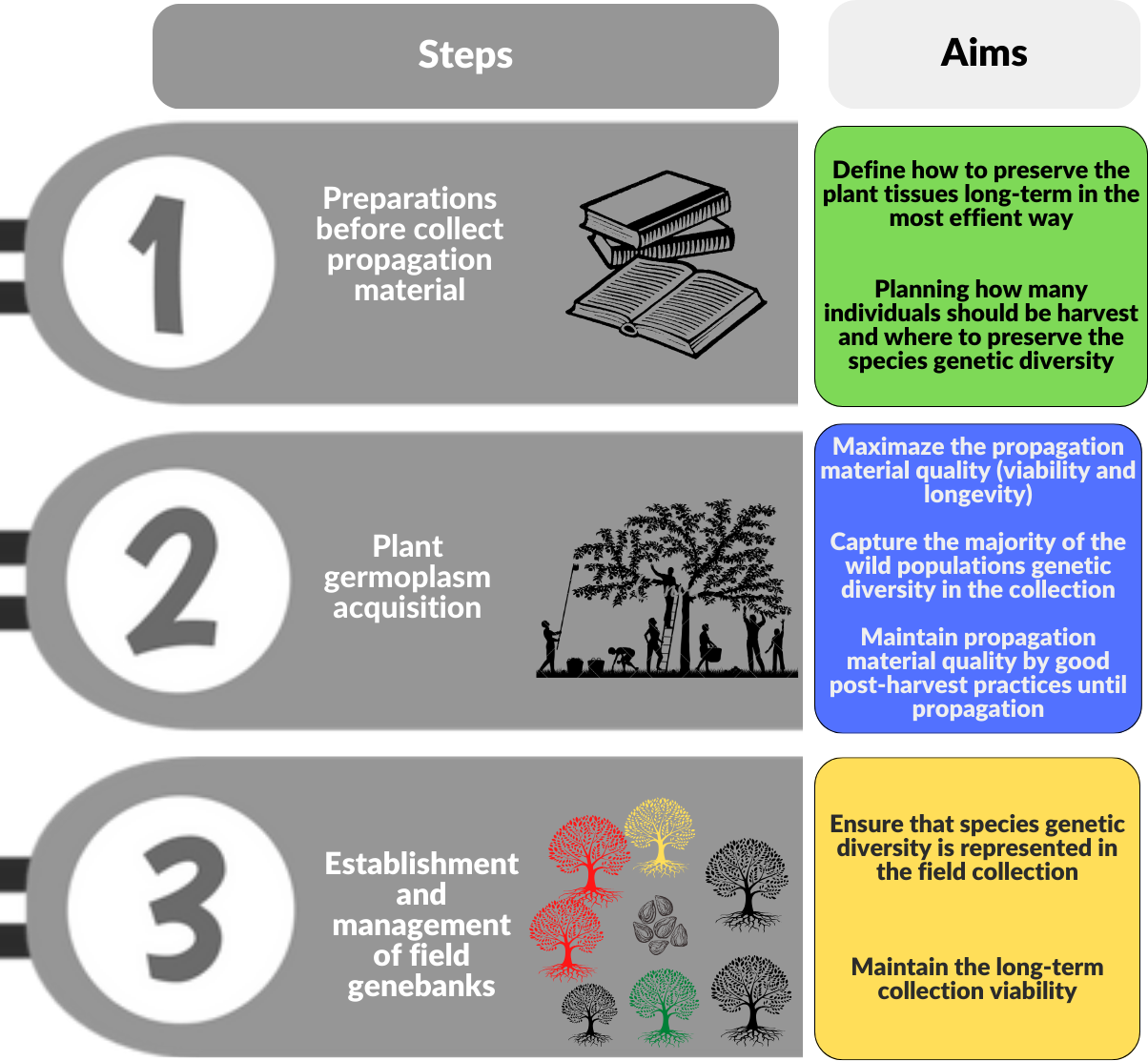


**Figure 1. Diagram flux of the general steps to build a field collection and aims.**

**Plant Germplasm Acquisition: harvest and post-harvest**

**Harvest propagating material**

Once the sampling sites, minimum number of individuals, and populations have been defined, the next step is to acquire the germplasm using procedures that maximize quality and genetic diversity (7). It is crucial to handle the propagating material properly in the field to ensure high viability and longevity, and to document it during collection to guarantee its survival until it reaches the conservation site. Plant species can be propagated through seeds, whole plants (e.g., seedlings), or plant parts (e.g., cuttings or tubers). Seeds are recommended as the propagation material because harvesting them has less impact on population growth rates, and they are more compact for transport and storage (15). For maximum germination capacity and longevity, always harvest fully ripe fruits, as these traits increase with seed maturity (7,21). For exceptional species, cuttings, rescued seedlings, or buds (tubers and rhizomes) may be used, depending on the species' biology. Vegetative propagules should be wrapped in moistened sterile cotton or paper, sealed in a plastic bag, and transported in a cool box to maintain moisture and cool temperatures (12). For a detailed information on the harvest and storage of propagules see (22–25).

To enhance genetic diversity, propagating material should be collected across the full range of phenotypic and temporal variation within a population. This involves taking samples from large and small maternal plants, as well as from the edges and center of a population (2,26). Additionally, collecting material over multiple years, rather than in a single season, captures diversity from individuals that flower under varying environmental conditions (27). Because propagation success can vary among populations and across years, as illustrated in the jaborandi case study, this strategy also increases the likelihood of establishing a higher number of plants per maternal line in the collection.

Depending on the conservation effort's aims, the population size, and the type of genebank, seeds can be collected at the population or individual level. Collecting fruits at the population level is faster, but the number of seeds per individual must be balanced (9). For small populations (<20 individuals) or endangered species, maintaining separate maternal lines allows flexibility in balancing family lines during reintroductions and prevents genetic issues such as inbreeding, genetic drift, and artificial selection (2,28).

Accurate documentation during sample collection is crucial for identification, characterization, and subsequent utilization. Specific formats should be used to gather and document collection data (9). Essential field information includes the accession number, the identification of the plant at the lowest possible taxonomic level, the number of individuals sampled, location details (place, province, and country), geographic coordinates, altitude, habitat, collector names, and the collection date (9). For population-level collections, assign a single accession number to all fruits harvested from a single plant population per harvest event. Store these fruits together in paper or cloth bags (for dry fruits) or plastic bags (for freshly harvested fruits), keeping them separate by population. At the individual level, keep seeds from each mother plant together as a single accession and separate them from those of other mother plants, even if they are from the same population (9). Accession numbers enable the tracking of the plant source from seed collection to use (e.g., reintroduction or field genebank establishment), so they must be maintained carefully throughout all stages of the germplasm bank.

**Post-harvest**

Effective post-harvest handling maintains the longevity of the propagules, prevents contamination, and ensures that propagation materials arrive at the seed bank or propagation facility in good condition (7). For practical post-harvest guidelines for seeds and vegetative propagules, see (29,30). In brief, post-harvest practices include cleaning the seeds to remove those that are empty, poorly developed, or infested with insects, and drying them to 50% equilibrium relative humidity for desiccation-tolerant seeds. Desiccation-sensitive seeds, however, should maintain their moisture content (7). Cuttings and seedlings should be kept wrapped in sterile cotton or a suitable alternative and placed in perforated plastic bags to allow air circulation until plant propagation (8). Additionally, the cut ends of cuttings should be covered with parafilm strips to minimize moisture loss (7).

The time between propagation material harvest and transfer to the seed bank or propagation facility should be minimized to ensure successful conservation, high germination percentages, and seedling vigor (7). This is particularly important for species that produce seeds and vegetative propagules, which are desiccation-sensitive and do not maintain viability for long. According to the Millennium Seed Bank Partnership (31), the period between collecting plant material, post-harvest handling, and either storage in seed banks or propagation for field planting should not exceed four weeks.

**Establishment and management of *inter situ* collections**

**Plant propagation and sapling production**

Plants are propagated in nurseries rather than direct sowing in the field to prevent pests and diseases, thereby increasing the likelihood of successful establishment (32). Indeed, the high acclimation success of jaborandi saplings supports the importance of the nursery to successful plant propagation. The controlled environment in nurseries enables researchers and practitioners to manage germination, growth, and survival rates, thus refining sampling strategies (14). Throughout nursery propagation, the source of propagation by accession number must be maintained, and saplings must be labeled prior to field allocation to allow for the traceability of maternal lines (2).

Understanding the requirements for seed germination is critical to maximizing propagation success. The naturally occurring temperature and soil moisture conditions during the growing season can be replicated in a nursery setting to optimize germination (33). However, germination is often constrained by seed dormancy. Approximately 50% of wild plants exhibit dormancy upon reaching maturity, requiring specific environmental cues to break it, such as fluctuating temperatures, winter conditions, or dry periods (34). Therefore, determining whether a species is dormant and how to alleviate dormancy is fundamental to successful propagation. For detailed guidelines on assessing dormancy and methods to break it, see (34–36).

**Establishment of *inter situ* collections**

The establishing and management of field genebanks incurs significant costs and risks, therefore careful planning is essential for successful implementation and operation (37,38). Crucial aspects include selecting appropriate planting sites, planning spatial distribution, determining the number of individuals per maternal line, ensuring accurate documentation and labeling, and employing appropriate cultivation practices (2,7).

*Inter situ* collections require enough space to accommodate accessions representing the necessary diversity for conservation purposes, while mitigating environmental stresses such as weather, disease, and pests (7,37). Planting sites can differ between *inter situ* collections according to the: 1) restoration of degraded habitats using threatened species and/or increasing the current distribution area of these species; and 2) long-term preservation of a representative genetic diversity and evolutionary potential of the species, and providing propagation materials to support *in situ* conservation efforts (37,39). The former approach is known as “*inter situs*” or “conservation introductions”, whereby new populations of a species are established in the wild under environmental conditions similar to those of the parent *in situ* populations (37). The later approach, known as “*quasi in situ*”, considers both neutral and adaptive genetic diversity to minimize the genetic and demographic threats associated with the creation of artificial plant populations (28,39).

The *inter situs* approach has been applied to degraded areas of low economic value outside the known range of species, such as abandoned agricultural land (40,41) or areas where species have become locally extinct (42,43). Selecting degraded areas simplifies jurisdictional procedures and improves accessibility, enabling the reintroduction of many species and facilitating horticultural and agricultural management to increase plant recruitment (41,42). In the *quasi in situ* approach, the planting areas must match the environmental conditions of the natural locations of the populations to maintain adaptive genetic diversity. Populations representing different eco-geographical regions or habitats must be isolated to reduce the likelihood of exogamic depressions (39).

Like other living collections, adequate spacing and plant density can reduce intraspecific competition, as well as disease and pest infestations, in *inter situ* collections (38). To preserve genetic integrity, conspecifics from different populations (congenerics) should be sufficiently separated to avoid gene flow and minimize unintended hybridization and exogamic depression (2). A planned spatial distribution improves the efficiency of space use and collection management. The exact location of each accession must be clearly defined and georeferenced, and individuals must be labelled with data, field accession numbers, and common names during the establishment phase (7). Field maps are essential for planning purposes and serve as backups for field labels, which can easily be lost or destroyed. Thus, prepare and regularly update these maps (7).

The number of individuals per accession or maternal line should be defined by balancing the need to maintain genetic diversity, maximize the conservation value of the collection, optimize the use of available space, and account for economic constraints (38). A minimum number of maternal lines is required to adequately represent the species’ genetic diversity, while maintaining multiple individuals per line enhances conservation value and promotes long-term sustainability. This approach facilitates reproduction and ensures the availability of material for reintroduction or restoration efforts (2,38).

The number of maternal lines needed to capture representative genetic diversity is determined during the sampling design stage. In contrast, the number of individuals per line depends on the type of propagation material, the genebank system, and spatial limitations. Conventional seed bank standards recommend storing at least 1,500–2,000 seeds per accession (2), whereas field and in vitro collections typically maintain 3–20 replicates per accession, although no formal standards exist for these systems (7).

Additionally, the number of individuals per maternal line may be adjusted based on adult establishment rates, available space, and species growth form. Notably, recommendations from conservation translocation studies refer to the total number of individuals introduced (i.e., founder population size), not to replication within maternal lines, and indicate that at least 300–500 founders are typically required to improve establishment success and promote plant recruitment (44,45).

**Management and regeneration of** ***inter situ* collections**

Plants in *inter situ* collections are under less intensive management compared to *ex situ* living collections (38,39,42). This management often involves intensive initial restoration and agricultural practices, such as pest and disease control, irrigation, soil amendments, and weeding, to establish the plants. However, the aim is to gradually reduce these interventions and allow the reintroduced species to become self-sustaining (39). Natural regeneration is a key objective of *inter situ* collections as it helps to maintain genetic diversity over time (46). Nevertheless, regular monitoring of genetic diversity is essential to ensure the integrity of the collection and to determine whether additional plant material needs to be reintroduced (2,7).

If the number of individuals per accession/maternal line falls below an unsustainable level, regeneration is necessary (38). Information on plant regeneration cycles, propagation procedures, plant labeling, and location maps should be documented in the genebank information system. Basic documentation includes the accession number, the sequential number of each plant within an accession, the georeferenced location of the regeneration, the type of propagation material used, the planting date, the survival rate, the management practices employed, the planting methods used, and the harvest dates (7).

**References**

1. Volis S. Conservation-oriented restoration – a two for one method to restore both threatened species and their habitats. Plant Divers. 2019 Apr;41(2):50–8. doi:10.1016/j.pld.2019.01.002

2. Center for Plant Conservation. CPC Best Plant Conservation Practices to Support Species Survival in the Wild. Escondido, CA.: Center for Plant Conservation; 2019. 245 p.

3. Pence VC, Meyer A, Linsky J, Gratzfeld J, Pritchard HW, Westwood M, et al. Defining exceptional species—A conceptual framework to expand and advance ex situ conservation of plant diversity beyond conventional seed banking. Biol Conserv. 2022 Feb;266:109440. doi:10.1016/j.biocon.2021.109440

4. Wyse SV, Dickie JB, Willis KJ. Seed banking not an option for many threatened plants. Nat Plants. 2018 Nov 2;4(11):848–50. doi:10.1038/s41477-018-0298-3

5. Martyn Yenson AJ, Sommerville KD, Guja LK, Merritt DJ, Dalziell EL, Auld TD, et al. Ex situ germplasm collections of exceptional species are a vital part of the conservation of Australia’s national plant treasures. PLANTS PEOPLE PLANET. 2024 Jan;6(1):44–66. doi:10.1002/ppp3.10421

6. Walters C, Richards CM, Volk GM. Genebank Conservation of Germplasm Collected from Wild Species. In: Greene SL, Williams KA, Khoury CK, Kantar MB, Marek LF, editors. North American Crop Wild Relatives [Internet]. Cham: Springer International Publishing; 2018 [cited 2025 Aug 29]. p. 245–80. Available from: http://link.springer.com/10.1007/978-3-319-95101-0_10 doi:10.1007/978-3-319-95101-0_10

7. FAO. Genebank Standards for Plant Genetic Resources for Food and Agriculture. Roma, Italy: FAO; 2014. 182 p.

8. Martyn Yenson, Amelia J., Offord, Catherine A., Meagher, Patricia F, Auld, Tony D., Bush, David. Plant Germplasm Conservation in Australia. 3rd ed. Canberra: Australian Network for Plant Conservation; 2021.

9. Di Sacco, A, Way, M, León Lobos, P, Suárez Ballesteros, C. I, Díaz Rodriguez J. V. Manual de recolección, procesamiento y conservación de semillas de plantas silvestres. Royal Botanic Gardens, Kew e Instituto de Investigación de Recursos Biológicos Alexander von Humboldt; 2020. 81 p.

10. Coates DJ, Byrne M, Moritz C. Genetic Diversity and Conservation Units: Dealing With the Species-Population Continuum in the Age of Genomics. Front Ecol Evol. 2018 Oct 23;6:165. doi:10.3389/fevo.2018.00165

11. Quiroga MP, Castello L, Quipildor V, Premoli AC. Biogeographically significant units in conservation: a new integrative concept for conserving ecological and evolutionary processes. Environ Conserv. 2019 Dec;46(4):293–301. doi:10.1017/S0376892919000286

12. Bozzano M, Jalonen R, Thomas E, Boshier D, Gallo L, Cavers S, et al., editors. Genetic considerations in ecosystem restoration using native tree species: the state of the world’s forest genetic resources - thematic study. Rome: FAO; 2014. 281 p.

13. Monteiro WP, Dalapicolla J, Carvalho CS, Costa Veiga J, Vasconcelos S, Ramos SJ, et al. Genetic diversity and structure of an endangered medicinal plant species (Pilocarpus microphyllus) in eastern Amazon: implications for conservation. Conserv Genet. 2022 Aug;23(4):745–58. doi:10.1007/s10592-022-01454-6

14. Hoban S. New guidance for ex situ gene conservation: Sampling realistic population systems and accounting for collection attrition. Biol Conserv. 2019 Jul;235:199–208. doi:10.1016/j.biocon.2019.04.013

15. Cochrane JA, Crawford AD, Monks LT. The significance of ex situ seed conservation to reintroduction of threatened plants. Aust J Bot. 2007;55(3):356. doi:10.1071/BT06173

16. Crossa J, Vencovsky R. Basic sampling strategies: theory and practice. In: Collecting Plant Genetic Diversity: Technical Guidelines - 2011 Update. Rome, Italy: Guarino, L. Ramanatha, Rao V. Goldberg, E.; 2011.

17. Hoban S, Volk G, Routson KJ, Walters C, Richards C. Sampling Wild Species to Conserve Genetic Diversity. In: North American Crop Wild Relatives. Greene, S., Williams, K., Khoury, C., Kantar, M., Marek, L.; 2018.

18. Kashimshetty Y, Pelikan S, Rogstad SH. Effective seed harvesting strategies for the ex situ genetic diversity conservation of rare tropical tree populations. Biodivers Conserv. 2017 Jun;26(6):1311–31. doi:10.1007/s10531-017-1302-3

19. Parra-Quijano M, Iriondo JM, Lamas ET. Strategies for the collecting of wild species. In: Collecting Plant Genetic Diversity: Technical Guidelines - 2011 Update. Roma, Italy: Guarino L, Ramanatha Rao V, Goldberg E; 2011.

20. Rosenberger K, Schumacher E, Brown A, Hoban S. Proportional sampling strategy often captures more genetic diversity when population sizes vary. Biol Conserv. 2021 Sep;261:109261. doi:10.1016/j.biocon.2021.109261

21. Hong TD, Ellis RH. A protocol to determine seed storage behavior. Rome, Italy: International Plant Genetic Resources Institute; 1996. 64 p. (IPGRI Technical Bulletin; 1).

22. Cochrane JA, Crawford AD, Errington G, Cuneo P, Viler M, Wood JA, et al. Seed and vegetative material collection. In: Plant Germplasm Conservation in Australia: Strategies and Guidelines for Developing, Managing and Utilising Ex Situ Collections. 3rd ed. Canberra: Amelia J. Martyn Yenson, Catherine A. Offord, Patricia F. Meagher, Tony D. Auld, David Bush, David J. Coates, Lucy E. Commander, Lydia K. Guja, Sally L. Norton, R.O. Makinson, Rebecca Stanley, Neville Walsh, Damian Wrigley, Linda Broadhurst; 2021.

23. Zahawi RA, Holl KD. Evaluation of different tree propagation methods in ecological restoration in the Neotropics. In: Genetic Considerations in Ecosystem Restoration Using Native Tree Species. Bozzano, M., Jalonen, R., Thomas, E., Boshier, D., Gallo, L., Cavers, S., Bordacs, S., Smith, P., Loo, J.; 2014. p. 85–96.

24. Way, Michael. Techniques and key issues in collecting crop wild relatives. In: Plant Genetic Resources: a review of current research and future needs. Kew, UK: Dulloo, M. Ehsan; 2021. p. 155–84. doi:10.19103/AS.2020.0085.08

25. Santos F, Cavalcante A, Cardoso A, Caldeira Júnior C, Carvalho Neto C, Escobar D, et al., editors. Guia de coleta de sementes e protocolos de germinação: espécies de interesse para conservação das cangas de Carajás [Internet]. ITV; 2023 [cited 2025 Aug 7]. Available from: https://www.itv.org/wp-content/uploads/2023/10/GuiaGerminacao_Carajas_20231013_rgb.pdf doi:10.29223/BOOK.Vale.Bioma.2023.01

26. Basey AC, Fant JB, Kramer AT. Producing native plant materials for restoration: 10 rules to collect and maintain genetic diversity. Native Plants J. 2015 Mar 1;16(1):37–53. doi:10.3368/npj.16.1.37

27. Griffith MP, Clase T, Toribio P, Piñeyro YE, Jimenez F, Gratacos X, et al. Can a Botanic Garden Metacollection Better Conserve Wild Plant Diversity? A Case Study Comparing Pooled Collections with an Ideal Sampling Model. Int J Plant Sci. 2020 Jun;181(5):485–96. doi:10.1086/707729

28. Zinnen J, Broadhurst LM, Gibson-Roy P, Jones TA, Matthews JW. Seed production areas are crucial to conservation outcomes: benefits and risks of an emerging restoration tool. Biodivers Conserv. 2021 Apr;30(5):1233–56. doi:10.1007/s10531-021-02149-z

29. MSBP. Post-harvest handling of seed collections. Kew, UK: Royal Botanic Gardens, Kew; 2022. (Technical Information Sheet). Report: 4. Located at: http://brahmsonline.kew.org/Content/Projects/msbp/resources/Training/04-Post-harvest-handling.pdf

30. Offord CA, Mills E, Percival J, Shade A, Turner SH, Viler M, et al. The role of the plant nursery in ex situ conservation. In: Plant Germplasm Conservation in Australia: Strategies and Guidelines for Developing, Managing and Utilising Ex Situ Collections. 3rd ed. Camberra, Australia: Amelia J. Martyn Yenson, Catherine A. Offord, Patricia F. Meagher, Tony D. Auld, David Bush, David J. Coates, Lucy E. Commander, Lydia K. Guja, Sally L. Norton, R.O. Makinson, Rebecca Stanley, Neville Walsh, Damian Wrigley, Linda Broadhurst; 2021.

31. Royal Botanic Gardens, Kew. The Millennium Seed Bank Partnership (MSBP) Seed Conservation Standards. [Internet]. Royal Botanic Gardens, Kew.: Royal Botanic Gardens, Kew.; 2014. Report. Available from: https://msb.rbge.org.uk/sites/default/files/MSBP%20Seed%20Conservation%20Standards.pdf

32. Instituto Forestal (Chile), Quiroz Marchant I, Chung Guin-po P, García Rivas E, González Ortega MP, Soto Guevara H. Vivero forestal: producción de plantas nativas a raíz cubierta [Internet]. INFOR; 2009 [cited 2024 Oct 8]. Available from: https://bibliotecadigital.infor.cl/handle/20.500.12220/17366 doi:10.52904/20.500.12220/17366

33. Escobar DFE, Rubio De Casas R, Morellato LPC. Many roads to success: different combinations of life‐history traits provide accurate germination timing in seasonally dry environments. Oikos. 2021 Nov;130(11):1865–79. doi:10.1111/oik.08522

34. Baskin CC, Baskin JM. Seeds: ecology, biogeography, and evolution of dormancy and germination. 2nd edition. San Diego, CA: Elsevier/AP; 2014.

35. Kildisheva OA, Dixon KW, Silveira FAO, Chapman T, Di Sacco A, Mondoni A, et al. Dormancy and germination: making every seed count in restoration. Restor Ecol. 2020 Aug;28(S3). doi:10.1111/rec.13140

36. MSBP. Germination testing: environmental factors and dormancy-breaking treatments [Internet]. Royal Botanic Gardens, Kew; 2022. (Technical Information Sheet). Report: 04. Available from: http://brahmsonline.kew.org/Content/Projects/msbp/resources/Training/04-Post-harvest-handling.pdf

37. Commander, L.E., Coates, David J., Broadhurst, L., Offord, C.A., Makinson, R.O., Matthes, M. Guidelines for the translocation of threatened plants in Australia. 3rd ed. Canberra: Australian Network for Plant Conservation; 2018. 168 p.

38. International Plant Genetic Resources Institute, Reed BM, Engelmann F, Dulloo ME, Engels JMM. Technical guidelines for the management of field and in vitro germplasm collections. Rome: IPGRI; 2004. 106 p. (IPGRI handbooks for genebanks; 7).

39. Volis S, Blecher M. Quasi in situ: a bridge between ex situ and in situ conservation of plants. Biodivers Conserv. 2010 Aug;19(9):2441–54. doi:10.1007/s10531-010-9849-2

40. Cochrane JA, Barrett S, Monks L, Dillon R. Partnering conservation actions. Inter situ solutions to recover threatened species in South West Western Australia. Kew Bull. 2010 Dec;65(4):655–62. doi:10.1007/s12225-010-9233-0

41. Kueffer C, Kaiser-Bunbury CN. Reconciling conflicting perspectives for biodiversity conservation in the Anthropocene. Front Ecol Environ. 2014 Mar;12(2):131–7. doi:10.1890/120201

42. Burney DA, Burney LP. Paleoecology and “inter-situ” restoration on Kaua’i, Hawai’i. Front Ecol Environ. 2007 Nov;5(9):483–90. doi:10.1890/070051

43. Monks L, Barrett S, Beecham B, Byrne M, Chant A, Coates D, et al. Recovery of threatened plant species and their habitats in the biodiversity hotspot of the Southwest Australian Floristic Region. Plant Divers. 2019 Apr;41(2):59–74. doi:10.1016/j.pld.2018.09.006

44. Silcock JL, Simmons CL, Monks L, Dillon R, Reiter N, Jusaitis M, et al. Threatened plant translocation in Australia: A review. Biol Conserv. 2019 Aug;236:211–22. doi:10.1016/j.biocon.2019.05.002

45. Maschinski J, Coates D, Monks L, Dillon R, Barrett S, Possley J, et al. Rare and Threatened Plant Conservation Translocations: Lessons Learned and Future Directions. In: Florentine S, Gibson-Roy P, Dixon KW, Broadhurst L, editors. Ecological Restoration [Internet]. Cham: Springer International Publishing; 2023 [cited 2026 Mar 2]. p. 287–322. Available from: https://link.springer.com/10.1007/978-3-031-25412-3_8 doi:10.1007/978-3-031-25412-3_8

46. Volis S. Complementarities of two existing intermediate conservation approaches. Plant Divers. 2017 Dec;39(6):379–82. doi:10.1016/j.pld.2017.10.005
